# Constructing a biodiversity terminological inventory

**DOI:** 10.1371/journal.pone.0175277

**Published:** 2017-04-17

**Authors:** Nhung T. H. Nguyen, Axel J. Soto, Georgios Kontonatsios, Riza Batista-Navarro, Sophia Ananiadou

**Affiliations:** National Centre for Text Mining, School of Computer Science, University of Manchester, Manchester, United Kingdom; University of Colorado, UNITED STATES

## Abstract

The increasing growth of literature in biodiversity presents challenges to users who need to discover pertinent information in an efficient and timely manner. In response, text mining techniques offer solutions by facilitating the automated discovery of knowledge from large textual data. An important step in text mining is the recognition of concepts via their linguistic realisation, i.e., terms. However, a given concept may be referred to in text using various synonyms or term variants, making search systems likely to overlook documents mentioning less known variants, which are albeit relevant to a query term. Domain-specific terminological resources, which include term variants, synonyms and related terms, are thus important in supporting semantic search over large textual archives. This article describes the use of text mining methods for the automatic construction of a large-scale biodiversity term inventory. The inventory consists of names of species, amongst which naming variations are prevalent. We apply a number of distributional semantic techniques on all of the titles in the Biodiversity Heritage Library, to compute semantic similarity between species names and support the automated construction of the resource. With the construction of our biodiversity term inventory, we demonstrate that distributional semantic models are able to identify semantically similar names that are not yet recorded in existing taxonomies. Such methods can thus be used to update existing taxonomies semi-automatically by deriving semantically related taxonomic names from a text corpus and allowing expert curators to validate them. We also evaluate our inventory as a means to improve search by facilitating automatic query expansion. Specifically, we developed a visual search interface that suggests semantically related species names, which are available in our inventory but not always in other repositories, to incorporate into the search query. An assessment of the interface by domain experts reveals that our query expansion based on related names is useful for increasing the number of relevant documents retrieved. Its exploitation can benefit both users and developers of search engines and text mining applications.

## Introduction

### Background

Biodiversity, a synergy between biology and diversity, is concerned with the study of the various levels of living entities on earth, from genes to ecosystems. It plays a central role in our daily lives, given its implications on ecological resilience, food security, species and subspecies endangerment and natural sustainability. To support the advancement of biodiversity research, several efforts aimed at storing and sharing biodiversity knowledge have been undertaken over the past few years, resulting in the creation of digital resources such as the Catalogue of Life, the Encyclopedia of Life, the Global Biodiversity Information Facility, and the Global Names Architecture.

The Catalogue of Life (CoL) [1] aims to document all known organisms on earth and currently contains more than 1.6 million species names. Its database is continuously being updated and peer reviewed for coherence and consistency by over 3,000 specialists. Similarly aiming to collect information on various organisms, the Encyclopedia of Life (EoL) [2] stores the names, descriptions and images of more than 1.3 million species, manually curated by more than 85,000 members. For each species, EoL provides information in the form of pictures, distribution maps, taxonomy tree, synonyms and common names in multiple languages. Meanwhile, the Global Biodiversity Information Facility (GBIF) [3] is a repository that enables institutions to publish their biodiversity data through common standards. The database currently consists of more than 640 million occurrence records pertaining to more than 1.6 million species. The Global Names Architecture (GNA) [4] is a free and open-source web-based infrastructure that aims to promote interoperability between a number of heterogenous biodiversity taxonomies. It is underpinned by the Global Names Index (GNI), a shared index of approximately 20 million species names corresponding to around two million taxa. Owing to the links between the different GNA-compatible taxonomies that GNI holds, additions or changes to one taxonomy are automatically propagated to the others.

With the huge number of species records that these manually curated resources contain, updating them is undoubtedly a time-consuming and laborious task. In addition, they cannot always capture the richness of terminological variation—a phenomenon that is however evident in content expressed in natural language, i.e., textual data such as biodiversity literature. An example of a resource holding vast amounts of text is the Biodiversity Heritage Library (BHL) [5], an open-access repository containing millions of digitised pages of legacy literature on biodiversity. Currently, BHL holds nearly 100,000 titles and over 170,000 volumes in many languages, accounting for a huge amount of textual content with over 150 million species mentions. The English subset alone, for instance, amounts to more than 24 million pages of text.

A species is typically designated by two names: a scientific name (in Latin) and a common, vernacular name. Scientific names typically follow Linnaeus’ binomial nomenclature [6, 7], which makes use of (1) the *generic* name or genus, which always appears with its initial letter in uppercase, and (2) the *specific* name or epithet. The species name *Panthera leo*, for example, has *Panthera* as its genus and *leo* as its epithet. However, there are cases that require further specification using names of a sub-genus, or a sub-species, which produces longer names. When a sub-genus is included in a scientific name, it must appear—enclosed in brackets—between the generic name and the epithet, and begin with an uppercase letter. For instance, *Cambarus (Puncticambarus) aldermanorum* (a type of crayfish) belongs to the *Cambarus* genus, under which taxonomists have created the subgenus *Puncticambarus* so that they can track affinities within the group. In practice, the sub-genus can be omitted. Meanwhile, sub-species names are written in lowercase and appended to the binomial name as in the examples *Strix nebulosa lapponica*—a sub-species of *Strix nebulosa* (great gray owl), *Panthera leo* ssp. *persica*—a sub-species of *Panthera leo* (lion) and *Areca insignis* var. *insignis*—a type of palm tree. A scientific name becomes more complex with the inclusion of its authority fields (i.e., the name of the first person to publish it and the year that it was coined), which can be written in different formats. For example, biologists can use *Panthera leo* (Linnaeus, 1758), *Panthera leo* Linnaeus, 1758 or *Panthera leo* Linnaeus 1758 to refer to the same species. Another challenge arises when biologists indicate genus or sub-genus by using only the initial character, such as in *C. (P.) aldermanorum* and *A. insignis*. It should be noted that such abbreviations lead to ambiguity since the initial character can represent a number of generic names. For instance, *A. insignis* can have as its full form either *Areca insignis* (palm) or *Ardea insignis* (white-bellied heron).

One of the most important tasks in the biodiversity informatics domain is the linking of a species name to the other names of the taxon it stands for [8]. Thus, many name linking services have been proposed, including GNA [4] and TaxaMatch [9]. GNA provides several online services to parse and normalise scientific names containing orthographic variations, mispellings or abbreviations. It has been integrated into BHL to facilitate indexing of optical character-recognised (OCR) documents based on scientific names. TaxaMatch, meanwhile, is a parser that is capable of decomposing a name into its semantic components, thus allowing the matching of genus and epithet names. These services are, however, focussed on linking only scientific names, and do not take into consideration vernacular ones. Without linking scientific names to vernacular names and vice versa, search systems such as the BHL could suffer from suboptimal recall [10]. For instance, with *Panthera leo* as a query term, the BHL search engine returned only 14 relevant English articles, whereas its vernacular name “lion” retrieved more than 100. Based on this example, more than 80% of articles can be potentially overlooked by the system if it does not take into account variants of a user-specified query term.

In this paper, we describe our text mining-based approach to the automatic construction of a biodiversity terminological inventory. Specifically, we demonstrate how distributional semantic models (DSMs) were applied on a large-scale textual resource, i.e., the English subset of BHL, in order to derive semantically related names. Semantic relatedness indicates how much two terms are related by any kind of taxonomical or functional relation [11], which in the context of this work could refer to scientific and vernacular species names denoting the same taxon, or other species related by, e.g., the family or habitat they share. In the process, we performed a comparative evaluation of various types of DSMs, which allowed us to select the model that performs the best on the task of identifying semantically related species names. To the best of our knowledge, this is the first study to comprehensively and comparatively investigate the use of various DSMs for compiling a terminological inventory. Furthermore, our work is the first attempt to automate the construction of such a resource for the biodiversity domain.

In order to demonstrate the usefulness as well as the advantages of the resulting term inventory compared to other biodiversity repositories, we developed a visual search interface that employs the inventory to suggest semantically related species names based on a measure of relatedness to the initial query. Although not within the scope of this paper, taxonomy curation could be another application of our inventory, in which existing taxonomies are populated in a semi-automatic manner, i.e., with a user-in-the-loop manually validating automatically suggested names.

### Related work

A number of tools have been developed to facilitate the text-based analysis of taxonomic names. On the one hand are tools for the fundamental task of recognising names within text, such as the dictionary-based TaxonFinder [12], Linnaneus [13] and OrganismTagger [14]; the machine learning-based NetiNeti [15]; and hybrid systems such as TaxonGrab [16] and SPECIES [17]. On the other hand are name-linking tools which are aimed at resolving taxonomic names mentioned in text to their preferred names, accomplished at three various levels: (1) orthographic or spelling, i.e, linking misspelt names to the correct ones; (2) nomenclature, i.e., taking into account scientific names with or without author, date and annotations; and (3) semantic, i.e., taking into account both scientific names and vernacular names. Several tools addressing the task at the first and the second levels are TaxaMatch [9], Taxonomic Name Resolution Service (TNRS) [18], gnaparser—a GNA service [4] and BiOnym [19]. TaxaMatch is a pipelined-based approach for linking misspelt scientific names of species to the correct ones. It employs a name parser to firstly decompose a scientific name into its semantic components (i.e., genus, epithet and species authority). It then uses a hybrid string matching method to compute a similarity score between the constituent components of a source and a target scientific name. TNRS [18] is a web-based application that integrates a modified version of TaxaMatch for linking scientific names of plants across different taxonomies. Experiments demonstrated that TNRS substantially increased the overlap between two taxonomic resources (i.e., the Integrated Taxonomic Information System and the National Center for Biotechnology Information taxonomic database) when compared to exact string matching. Similarly to Boyle et al. [18], Patterson et al. [8] used a variation of the TaxaMatch algorithm to link scientific names of species across multiple taxonomies. The proposed algorithm was shown to better address challenging cases for fuzzy string matching algorithms such as erroneous alignment of homonym terms, i.e., terms that have different meanings but share the same spelling. BiOnym [19] is another taxon name matching system that allows users to select their preferred list of names to be incorporated into the system.

Considering that our proposed work is capable of detecting scientific and vernacular names that are taxonomically (i.e., referring to the same taxon) or functionally related (i.e., sharing the same family or habitat), it can be viewed as a method for linking names at the semantic level, applied to the automatic construction of specialised terminological resources. Existing approaches to this task can be coarsely classified into string-based, knowledge-based and distributional semantic methods. String-based methods exploit surface features (e.g., character *n*-grams, bi-grams, prefixes, suffixes etc.) to compute a similarity score between two terms. Thompson et al. [20] used a fuzzy string matching method as part of a larger text mining pipeline to construct the BioLexicon, a large-scale lexicon of biomedical terms. Their proposed pipeline consists of several components, including: a) automatic term extractors [21] and named entity recognisers [22], which extract mentions of terms and named entities in text; b) a fuzzy string matching method that identifies term variants of the same concept; and c) syntactic and semantic parsers used to automatically acquire the grammatical and semantic frames within which concepts appear. The BioLexicon, which indexes approximately 1.8 million terminological variants with over 2 million synonymy relations, has been proven useful for a number of applications (e.g., lemmatisation of biomedical text, information retrieval and information extraction [23, 24]). Tsuruoka et al. [25] presented a string matching method to capture synonyms between gene/protein names. They used logistic regression to learn a novel string similarity measure based on a gene/protein name dictionary extracted from the Unified Medical Language System (UMLS) [26]. Experimental results showed that their approach outperformed string matching methods that are based on existing similarity measures such as Jaro-Winkler [27] and SoftTFIDF [28].

While string matching methods can effectively identify synonyms that vary only at the orthographic level, they perform poorly when a name shares no orthographic similarities with its variants. Such cases are prevalent in the biodiversity domain where synonymous scientific and vernacular names typically have no common orthographic features (e.g., *Strix nebulosa* vs. great grey owl).

Going beyond capturing orthographic similarities, knowledge-based methods [29–32] leverage external knowledge resources, such as thesauri (e.g., UMLS [26]), ontologies (Gene Ontology [33]) or semantic networks of concepts (e.g., WordNet [34]), to estimate semantic similarity between two terms. In the biomedical domain, several approaches investigate the use of knowledge-based methods to compile or update term inventories. Wang et al. [31] used the Gene Ontology (GO) to compute the semantic similarity between biochemical or biological terms while Lee et al. [32] exploited the SNOMED-CT thesaurus to identify semantically similar clinical terms. A limitation of existing knowledge-based approaches is that their performance depends upon the coverage of manually curated knowledge resources, which may not be readily available for certain domains.

In contrast, distributional semantic models (DSMs) do not rely on hand-curated knowledge resources. They compute semantic relatedness based on domain- and language-independent models, which have demonstrated superior performance over knowledge-based approaches for the estimation of semantic similarity [35, 36]. DSMs encode the lexical context of a term into a vector representation and calculate term relatedness as the cosine similarity between term vectors. The lexical context is determined using count-based distributional semantics, i.e., by calculating the frequency of the words that occur within a term’s neighbourhood. Henriksson et al. [37] used a count-based DSM to construct a terminological inventory of clinical terms from a collection of electronic health records. Whilst count-based DSMs have been shown to work well when applied to single-word terms, they obtain suboptimal performance on multi-word terms, i.e., those comprised of multiple words. Previous studies have however observed that multi-word terms account for more than 90% of the terms indexed by specialised terminologies [37]. Thus, methods that accurately identify synonymous pairs of multi-word terms are crucial for the efficient construction of terminological resources. Examples of such methods include compositional DSMs, which employ a function of a multi-word term’s constituent words to encode it as a vector. Upon applying different DSMs on two medical historical archives, Thompson et al. [38] demonstrated that a compositional DSM obtained improved performance over standard count-based DSMs. The semantically related terms identified by their compositional DSM formed the basis of a terminological inventory of medical concepts, which was then integrated into a semantic search interface to allow users to explore the evolution of medical terms over time.

One disadvantage of count-based DSMs (compositional or not) is their underlying bag-of-words (BOW) approach for capturing lexical context, i.e., the reliance on raw co-occurrence frequencies between a term and its neighbouring words. Such representation of lexical context does not take into account word order and semantic information, since all neighbouring words are considered equally distant from a given term and are represented as an unordered bag-of-words collection [39].

In this article, we explore the use of more sophisticated DSM methods, i.e., prediction-based DSMs, to efficiently construct a terminological inventory of biodiversity terms. In contrast to count-based DSMs, prediction-based models exploit an embedded representation of words or phrases, which take into account the semantics and the order of the words. Baroni et al. [39] claimed that prediction-based models, e.g., continuous bag-of-words (CBOW) [40], are superior to count-based models [41]. However, recently, Levy et al. [42] demonstrated that the superior performance of prediction-based models results from certain selections of hyper-parameters rather than from the methods themselves. Since there are no conclusive studies yet establishing which of the two types of models is superior, we carried out experiments exploring both count-based and prediction-based models. For count-based models, we employed the standard model [43–45] and a compositional DSM, namely the basic additive model [46]. We then compared these models with prediction-based models, e.g., CBOW [40] and global vectors [47]. We demonstrate that prediction-based models perform better relative to the count-based and compositional DSMs. To the best of our knowledge, our work is the first to utilise prediction-based DSMs for the automatic compilation of a large-scale terminological resource.

## Methods

**Fig 1:**
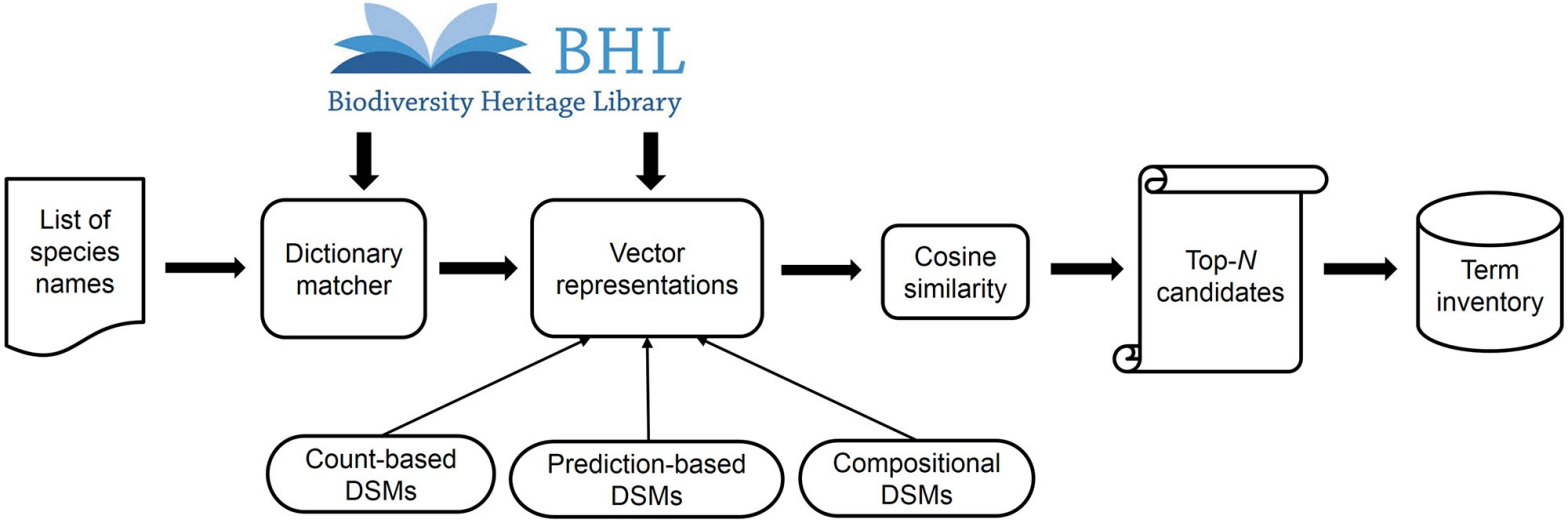
Framework for constructing our biodiversity terminological inventory.

Our workflow for constructing the term inventory is presented in Fig 1. Firstly, for the identification of terms in text, we compiled a dictionary that includes all the names available in CoL, EoL and GBIF, and then applied a string-matching method on the English subset of the BHL. It is worth noting that name disambiguation is not necessary at this step. Next, given the identified terms, we learn their vector representations using two different approaches, i.e., count-based and prediction-based, and for each of these main approaches we also apply the compositional version. These approaches compute vector representations for both single and multi-word terms. The cosine similarity between two vectors serves as an indicator of the corresponding terms’ semantic relatedness: the higher the cosine similarity, the more related the two terms are. We finally select the top-*N* candidates as the terms that are most semantically related to a given term.

We employ an in-house implementation of the standard count-based model [43–45]. The method applies basic pre-processing steps to normalise text contained in the BHL documents. Normalisation includes stop-word and part-of-speech filtering to remove prepositions and other common words, as well as lemmatisation, i.e., the mapping of a word to its base form (also known as lemma). A context vector is then created for each term, based on all normalised lexical units that occur within a symmetrical window of *w* words around the term. Each element (or dimension) of a context vector represents a lexical unit; it holds the calculated correlation—the log-likelihood ratio—between the corresponding lexical unit and the term. However, as this step produces high-dimensional context vectors that tend to be sparse, we maintain only the *d* most frequently occurring lexical units and discard the rest. The resulting context vectors thus consist of only *d* dimensions.

The count-based approach has been shown to efficiently compute the semantic similarity between single-word terms [43]. However, the performance of the model is known to decline when applied to longer sequences (e.g., phrases or sentences) [48]. In response, researchers have explored the use of approaches based on compositional distributional semantics that compute a word sequence’s lexical context as a function of the lexical context of its constituent words [46, 49]. In this work, we experiment with an existing compositional semantics model [46], namely the basic additive model (BAM). BAM computes the context distribution of a multi-word term as a linear combination of its component word vectors (previously computed using the standard count-based model). Given a multi-word term consisting of *K* words *z* = *w*_1_, ⋯, *w*_*i*_, ⋯, *w*_*K*_ and the context vectors of the *K* constituent words *v*(*w*_1_), ⋯, *v*(*w*_*i*_), ⋯, *v*(*w*_*K*_), we compute the combined vector *v*_*BAM*_(*z*) of *z* using the follow equation:

$$\begin{array}{c} v_{BAM}\left(z\right)=\frac{v(w_{1})}{∥v(w_{1})∥}+\cdots +\frac{v(w_{i})}{∥v(w_{i})∥}+\cdots +\frac{v(w_{K})}{∥v_{w_{K}}∥}, \end{array}$$ (1)

where ‖*v*(*w*_*i*_)‖ is the *L*2 norm of the context vector for the *i*^*th*^ word.

Prediction-based models, meanwhile, were conceived based upon the same principle as the count-based models. However, instead of purely counting words’ occurrences, prediction-based models learn vector representations by optimising an objective function based on the probability that a word co-occurs with the words within its neighbourhood or context. By doing so, the models can estimate the contexts in which the corresponding words tend to appear. Moreover, the resulting vectors usually have lower dimensionality than their counterpart count-based vectors. In this paper, we used prediction-based models following two well-known approaches: *continuous bag-of-words* (CBOW) [40] and *global vectors* (GloVe) [47]. The CBOW model is similar to the feed-forward neural network language model [50] which, has no hidden layer and whose projection layer is shared by all words. This model predicts a word by using the continuous context around that word. Meanwhile, GloVe optimises the likelihood of word probabilities based on context, to learn word representations as in CBOW but uses ratios of co-occurrence probabilities as the basis for learning.

It is worth noting that a compositional DSM can also be applied after learning single-word vectors using prediction-based DSMs. To this end, we encoded multi-word terms according to two different vector representations:

- *Multi-word vectors*. We introduced some slight modifications to the text by placing an underscore between words comprising multi-word terms (e.g., by turning “chipping sparrows” into“chipping_sparrows”). We then input the resulting text to the count-based model, CBOW and GloVe to directly learn vector representations for these multi-word terms.
- *Single-word vectors (BAM)*. After using count-based, CBOW and GloVe to learn single word vectors, we calculate a multi-word term vector by applying BAM, i.e., cumulatively adding the single-word vectors pertaining to a multi-word term’s individual components.

In summary, we have applied and evaluated six different distributional semantic models: *count-based*, *CBOW*, *GloVe*, and their corresponding compositional models which we shall refer to as *count-based-BAM*, *CBOW-BAM* and *GloVe-BAM*.

## Comparative evaluation

In order to evaluate the performance of the proposed methods, we conducted a series of experiments on species names from three categories: birds, mammals and plants. The performance of each proposed method was measured by using metrics such as top-*N* accuracy, recall and precision at *N* and mean average precision (MAP).

### Data preparation

We programmatically acquired the English subset of BHL by using its publicly available application programming interface (API) [51]. The API provides functions for retrieving the OCR text of each document as well as corresponding metadata, e.g., the document’s language and publication date. In this paper, we focus on a BHL subset consisting of pages whose language—according to the value of a BHL metadata field—is English. We note, however, that the value assigned to this language field is not always correct, leading to the inadvertent inclusion of pages which were actually written in other languages (e.g., German). The resulting corpus contains more than 24 million pages amounting to a total of around 49 gigabytes of data.

We were unable to find gold standard resources suitable for evaluating tools that link semantically related terms to a given species name. To support the evaluation of our DSMs, we utilised four taxonomies, i.e., the Catalogue of Life, the BirdLife Taxonomic Checklist, the Interagency Taxonomic Information System (ITIS) and the PLANTS Database, for the compilation of reference standard data sets containing semantic variants. In this work, for every preferred name *n*—the binomial name that is most commonly used to denote a certain species *s*—we define *semantic variants* as the set of names containing: (1) scientific names synonymous to *n*, and (2) vernacular names that denote *s*. In BirdLife, for instance, the scientific name *Actitis macularius* has two strict-sense synonyms, i.e., *Actitis macularia* and *Tringa macularia*, and one vernacular name, i.e., “spotted sandpiper”. *Actitis macularius* is the preferred name, and *Actitis macularia*, *Tringa macularia* and “spotted sandpiper” are its corresponding semantic variants (as shown in Table 1).

**Table 1:** Examples from the reference standard data set extracted from manually curated databases.

Databases	Preferred name	Semantic variants
BirdLife	*Actitis macularius*	*Actitis macularia*, *Tringa macularia*, spotted sandpiper
	*Tachycineta bicolor*	tree swallow
	*Eudyptula minor*	little penguin, blue penguin, fairy penguin
ITIS Mammals	*Ateles paniscus*	*Ateles ater*, black spider monkey
	*Rhinoceros unicornis*	*Rhinoceros indicus*, Indian rhinoceros
	*Cricetus cricetus*	*Cricetus frumentarius*, *Cricetus vulgaris*, common hamster
The PLANTS database	*Cissampelos pareira*	velvetleaf
	*Campsis radicans*	*Bignonia radicans*, *Tecoma radicans*, trumpet creeper
	*Abutilon theophrasti*	*Abutilon abutilon, Abutilon avicennae*, velvetleaf

It is worth noting that a vernacular name can denote multiple taxa. For example, in Table 1, “velvetleaf” is a vernacular name of both *Cissampelos pareira* and *Abutilon theophrasti*. Although “velvetleaf” is treated as a semantic variant of each of *Cissampelos pareira* and *Abutilon theophrasti*, the latter two should not be considered as the same taxon.

Two distinct reference standard data sets were compiled: one based on the manually curated BirdLife, ITIS and PLANTS taxonomies, and the other based on the Catalogue of Life (CoL) taxonomy, which was formed through the semi-automatic aggregation of many other taxonomies. In compiling the former, the following data was gathered: (1) names of 10,424 recognised birds from the BirdLife Taxonomic Checklist v8.0 [52]; (2) names of 9,044 mammals from the Interagency Taxonomic Information System (ITIS) [53]; and (3) names of 33,513 plants from the PLANTS database [54]. The data set based on CoL, meanwhile, consists of three subsets: names of 9,666 species from the “Aves” taxonomic class, names of 5,593 species from “Mammalia”, and names of 115,966 plants from “Magnoliopsida”.

We then selected preferred names that appear in both the BHL corpus and the reference standards. We retained only those that have at least one synonym or vernacular name according to the reference standards, and which appear in the BHL corpus frequently enough, i.e., at least 50 times. We finally randomly selected 500 preferred names for each of the bird, mammal and plant categories.

As mentioned above, scientific names of species come in various forms. From a computational perspective, it is a less interesting or trivial task to identify synonymous scientific names that differ from the binomial form only in terms of having the sub-genus or sub-species name. We thus retained in the reference standard only the scientific names that conform to the binomial nomenclature; those that include sub-genus and sub-species names as well as authority fields were not included in this evaluation, although they will be covered in our future work. For each of the bird, mammal and plant categories, the resulting reference standard consists of more than 1,000 unique names accounting for both preferred names and their variants, i.e., synonymous scientific and vernacular names. More of these details are summarised in Table 2. Names contained in the reference standard are provided in S1 Text.

**Table 2:** Frequencies of semantic variants in the reference standard data sets.

Category	Source	No. preferred names	No. of variants	Total names	Avg. no. of variants per preferred name
Birds	BirdLife	500	628	1128	1.26
	CoL	500	622	1122	1.24
Mammals	ITIS	500	629	1129	1.26
	CoL	500	635	1135	1.27
Plants	PLANTS	500	770	1270	1.54
	CoL	500	1195	1695	2.39

Our analysis of the reference standard data sets reveals that there are many semantic variants sharing identical component words with preferred names. Some of them have identical genus names since the species originated from the same genus in the taxonomy tree. For example, as shown in Table 1, *Cricetus cricetus* has semantic variants such as *Cricetus frumentarius* and *Cricetus vulgaris*, all sharing the same genus name *Cricetus*. Some of them have their specific epithet identical with the genus of the preferred name. For instance, two of the semantic variants of *Campsis radicans* are *Bignonia radicans* and *Tecoma radicans*, all having in common the name *radicans*. We show the frequency of such phenomena in Table 3. It can be seen that a large number of plant names (about 68% for PLANTS and 85% for CoL) have overlaps between the preferred names and their semantic variants, whilst this ratio is less than 29% in the case of bird and mammal names.

**Table 3:** Number of semantic variants that share genus and specific epithet names with the preferred names. No. of preferred names: the number of preferred species names that share a component name with at least one variant; No. of variants with shared genus: the number of variants that share the genus name with the preferred names; No. of variants with shared specific epithet: the number of variants that share the specific epithet name with the preferred names.

Category	Source	No. of preferred names with shared component name	No. of variants with shared genus	No. of variants with shared specific epithet
Birds	BirdLife	70	24	50
	CoL	114	30	89
Mammals	ITIS	135	102	65
	CoL	144	106	62
Plants	PLANTS	343	341	124
	CoL	426	697	193

### Evaluation parameters and metrics

In applying the six distributional semantic models that we selected, we defined the lexical context as the words surrounding a term within a window *w*, where *w* = 3. The resulting context vectors have a dimensionality of 150,000 and 300 for count-based and prediction-based models, respectively.

We used four different metrics to measure the performance of the DSMs, namely: top-*N* accuracy, precision and recall at *N*, and mean average precision (MAP). For a given term, a model generates a list of semantically related terms, ranked according to their cosine similarity. Rather than showing the performance obtained upon taking only the topmost candidate, we measure performance using the aforementioned metrics while taking top-*N* candidates, where *N* is set to values ranging from 1 to 20 in an incremental fashion. Top-*N* accuracy [55] is the ratio between the number of input terms whose top-*N* ranked candidates include at least one correct semantic variant, and the total number of terms in consideration. Precision and recall at *N* (precision@*N* and recall@*N*, respectively) are calculated in the standard manner: precision@*N* is the number of correctly identified semantic variants over *N*, whilst recall@*N* is the total number of correctly identified semantic variants out of the total number of semantic variants in the reference standard. Meanwhile, MAP [56] is a common metric used in information retrieval to evaluate ranked results. An advantage of MAP over the two previous metrics is that it measures precision at various levels of recall, hence eliminating the need to set a cut-off value *N*. In addition, MAP takes the ranked order of all candidates into account, whilst the other metrics consider the *N* topmost candidates as a set, i.e., without considering order.

### Results

This subsection describes the results of our DSM models when compared to semantic variants in reference standard data sets. Statistical tests applied to determine the significance of the performance-wise differences amongst the models are presented in the next subsection.

#### Birds

**Fig 2:**
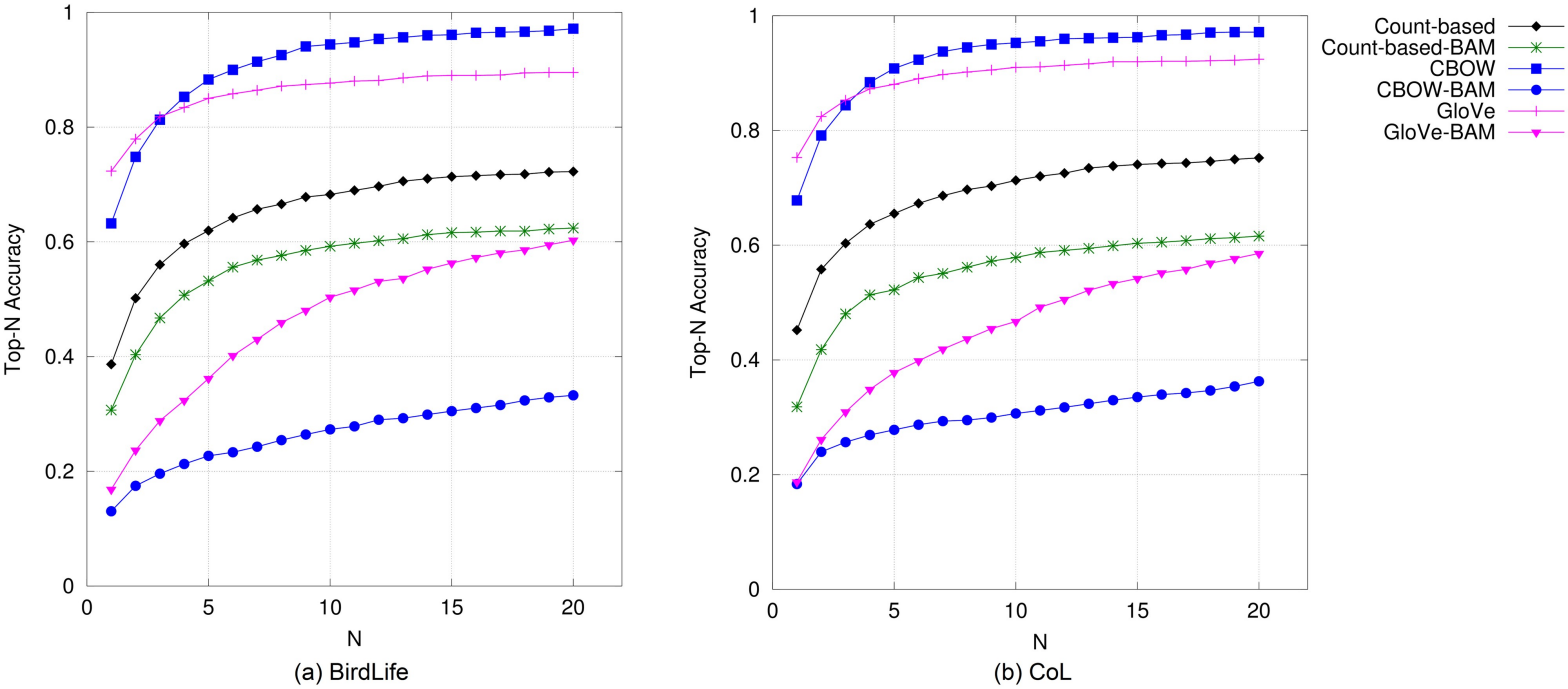
Top-*N* accuracy obtained by DSMs for bird names.

**Fig 3:**
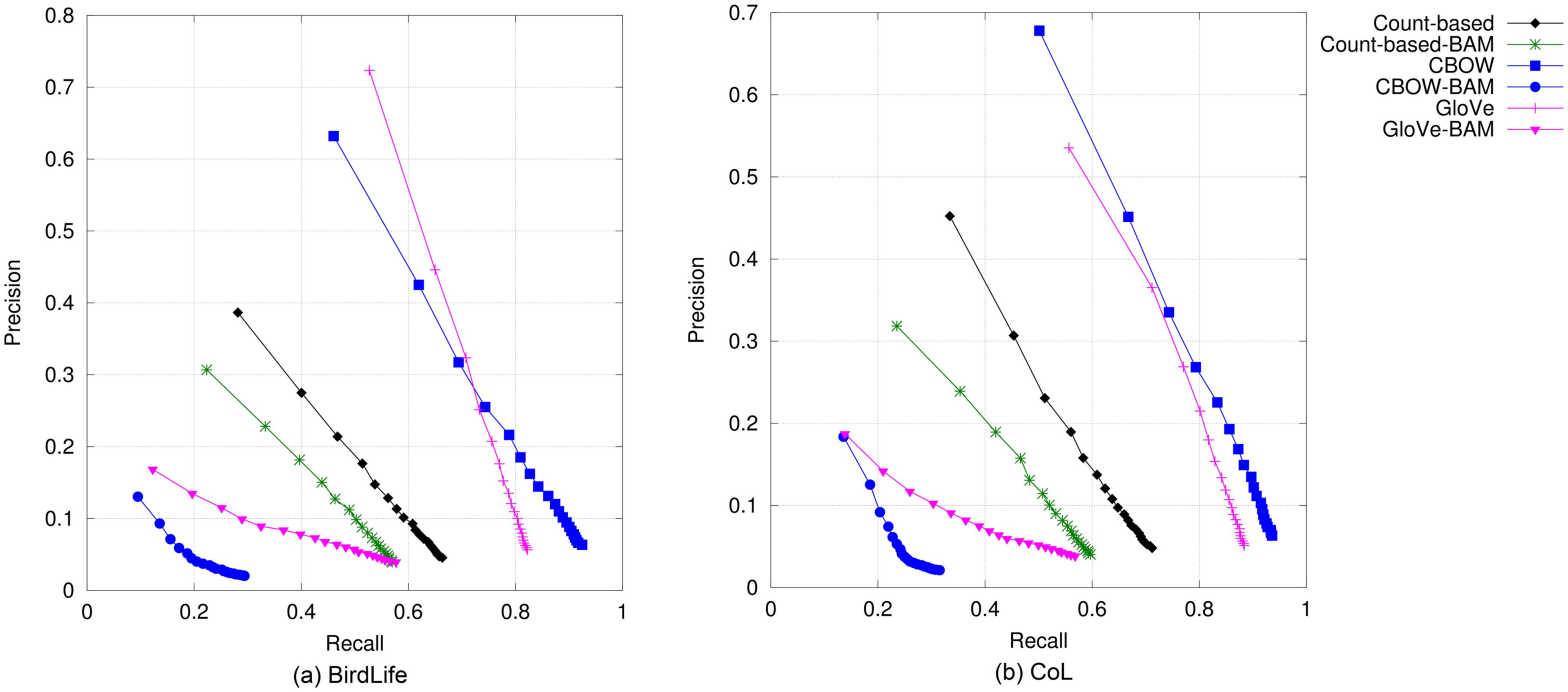
Precision@*N* versus recall@*N* obtained by DSMs for bird names when *N* is varied from 1 to 20.

The results on bird names are presented in Fig 2 (top-*N* accuracy) and Fig 3 (precision@*N* vs. recall@*N*). We can observe that CBOW and Glove obtain the best performance in terms of top-*N* accuracy, precision and recall when compared to all other DSM models. Results from GloVe are only slightly better than CBOW’s when only up to the top three candidates are considered, whilst CBOW is superior when the 4^*th*^–20^*th*^ topmost candidates are evaluated. It can also be observed that in this species category, the non-compositional vectors performed better than the compositional counterparts both for predictive-based and count-based models. A similar trend holds in the evaluation results according to MAP, which are presented in Table 4. As with the results using accuracy, precision and recall, non-compositional models performed better than compositional ones, with GloVe obtaining the best performance amongst all models. Between BirdLife and CoL bird names, the performance of each model is ranked quite similarly for all metrics.

**Table 4:** The Mean Average Precision (MAP) obtained by each method on bird, mammal and plant names. Bold numbers denote the highest scores obtained per general DSM type in each standard.

Approach	Model	Birds		Mammals		Plants	
		BirdLife	CoL	ITIS	CoL	PLANTS	CoL
Count-based	Count-based	**44.28**	**49.86**	**39.04**	**39.27**	38.79	36.24
	Count-based-BAM	34.15	36.90	30.54	30.25	**44.36**	**45.40**
Prediction-based	CBOW	69.56	73.34	52.17	53.12	**62.14**	51.49
	CBOW-BAM	13.28	16.86	19.46	19.08	49.03	**51.78**
	GloVe	**72.79**	**77.11**	**70.76**	**73.30**	28.30	33.77
	GloVe-BAM	22.86	24.06	27.44	27.82	46.71	46.65

#### Mammals

**Fig 4:**
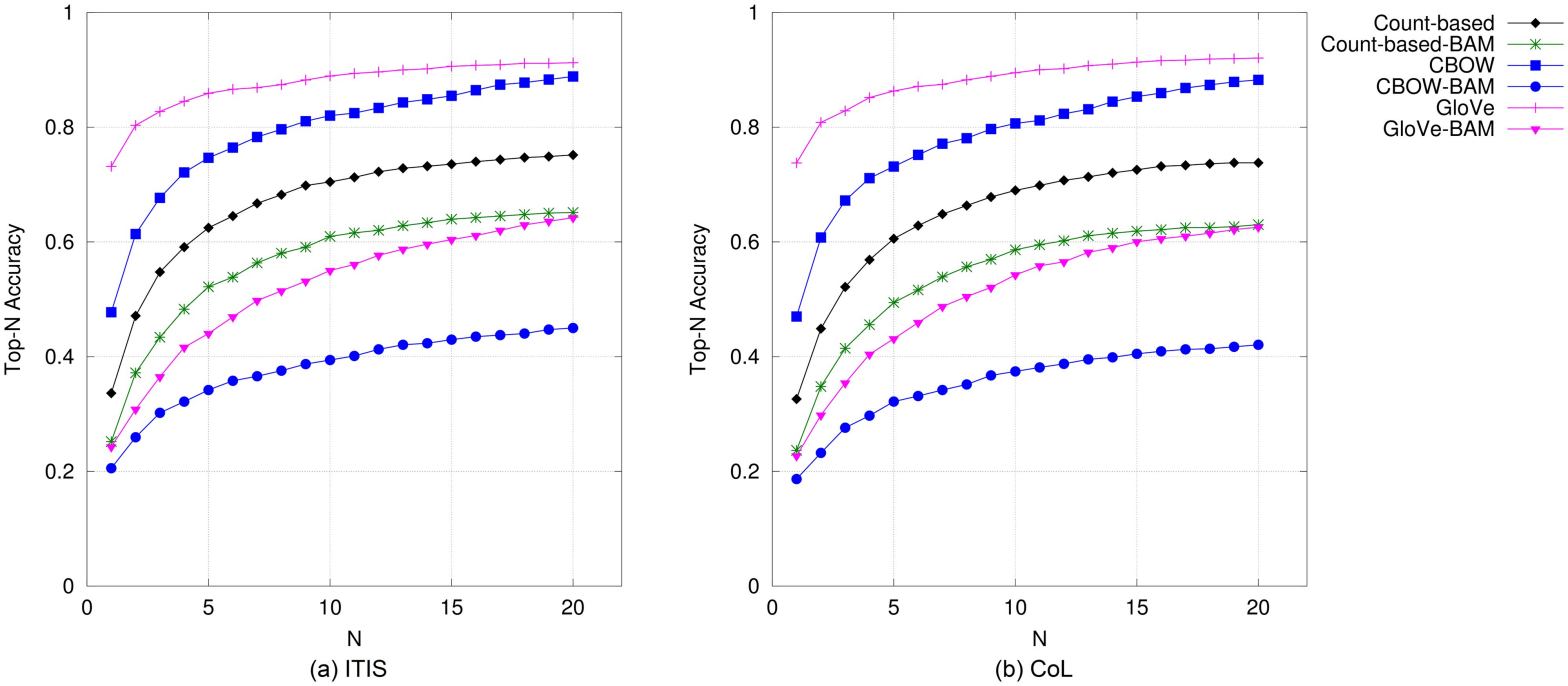
Top-*N* accuracy of DSMs for mammal names.

**Fig 5:**
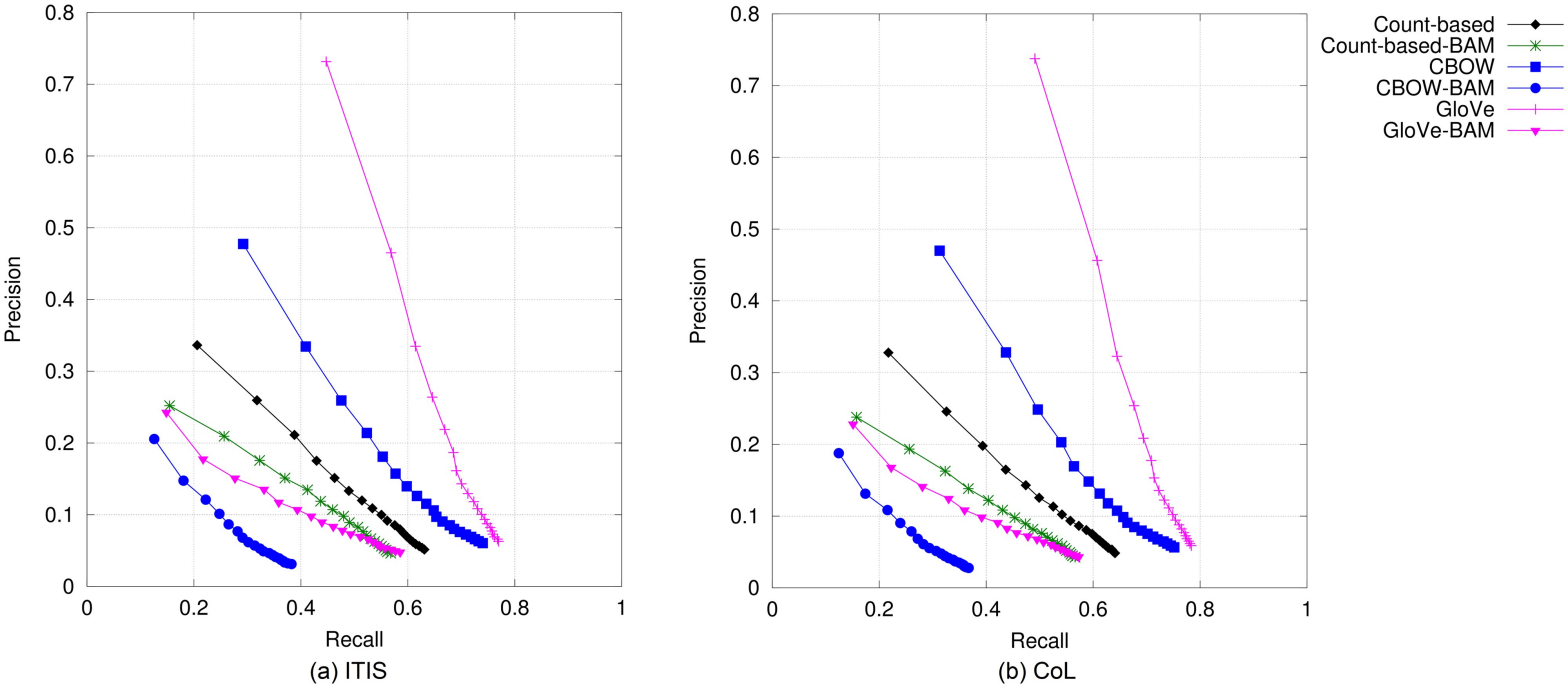
Precision@*N* versus recall@*N* obtained by DSMs for mammal names when *N* is varied from 1 to 20.

Top-*N* accuracy and precision-vs-recall curves for the six models are shown in Figs 4 and 5, respectively. Compared to the evaluation results on bird names, there are two minor differences. Firstly, GloVe obtained the best performance, closely followed by CBOW for every metric. Secondly, whilst count-based BAM still performs better than the prediction-based compositional models, performance gains over Glove-BAM are much smaller than in the case of bird names. In terms of MAP, our results, which are shown in the fourth and fifth columns of Table 4, reveal that GloVe is again the best performing model. The similar trends in the models’ performance for mammal and bird names were expected, given the similarities between the two reference standard data sets as depicted in Tables 2 and 3. Comparing the performance when evaluating against ITIS mammal names and that when using CoL, we can see only a small difference, i.e., about 0.2 to 2.5 percentage points.

#### Plants

**Fig 6:**
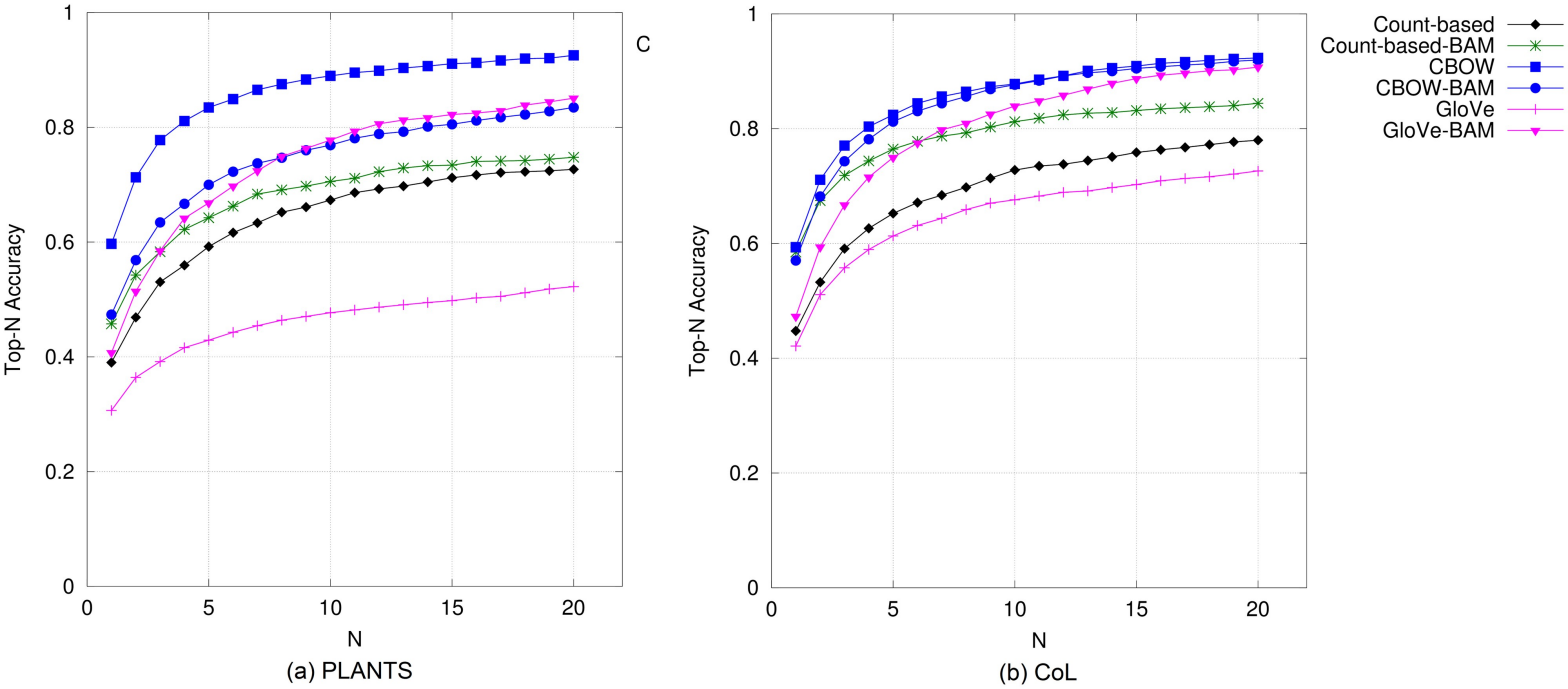
Top-*N* accuracy obtained by DSMs for plant names.

**Fig 7:**
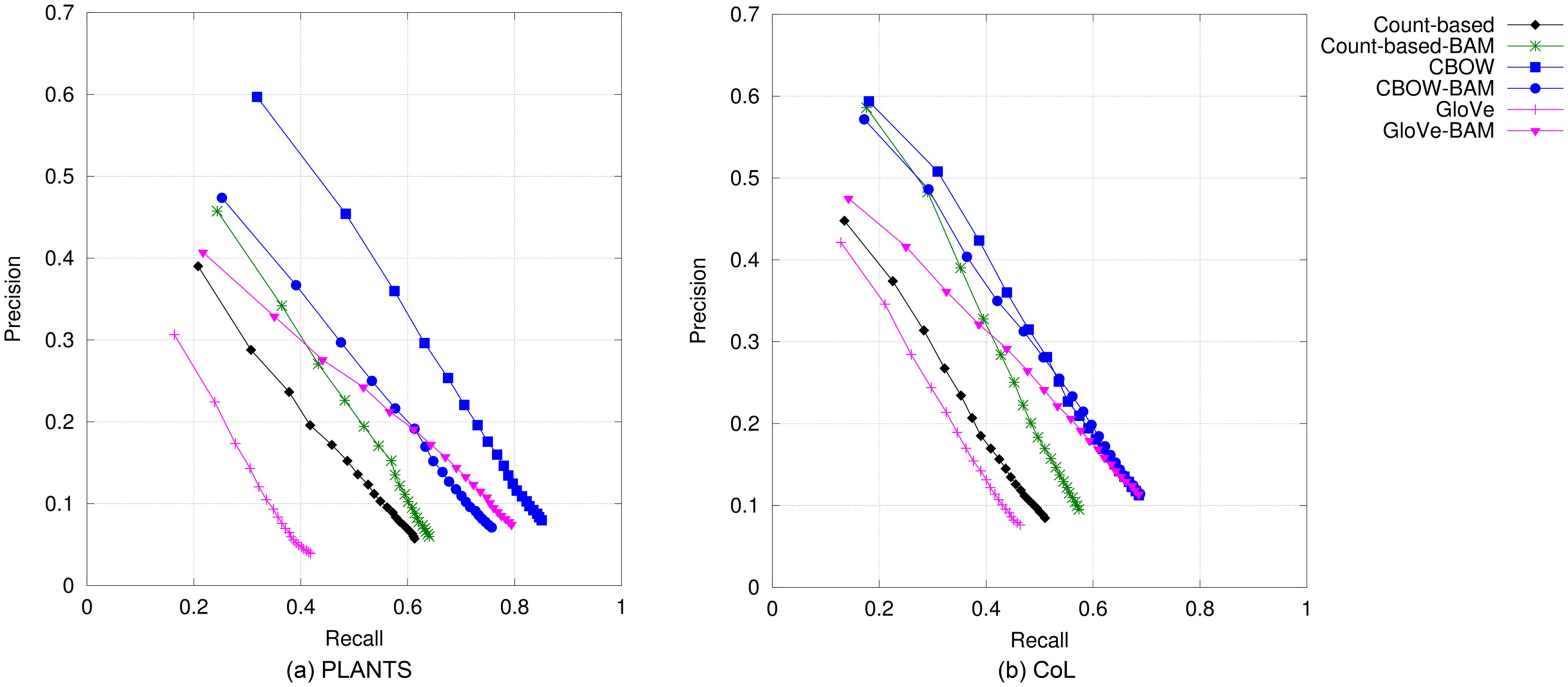
Precision@*N* versus recall@*N* obtained by DSMs for plant names when *N* is varied from 1 to 20.

Results on plant names based on top-*N* accuracy and precision/recall are illustrated in Figs 6 and 7, respectively. In comparison to results on bird and mammal names, the models performed differently for this species category. On the PLANTS database, CBOW performed the best amongst all DSMs, followed by all the compositional models. With CoL plant names, CBOW-BAM and CBOW are the best ones and also comparable to each other, with GloVe-BAM trailing right behind. The performance of standard GloVe in this category is unexpectedly poor for all metrics. The results for this category based on MAP also show the same pattern as in those based on the other metrics (as presented in the last two columns of Table 4).

### Discussion

We note that our DSMs obtained similar performance on bird and mammal names but quite different results on plant names. Amongst the two count-based models, the standard one obtained the best scores on both sets of reference standards for bird and mammal names, but performs worse than the compositional model on plant names. Regarding prediction-based ones, GloVe shows the same trend, i.e., it is better than CBOW in the case of bird and mammal names but performed poorly on plant names. The compositional prediction-based models exhibited optimal performance on plant names. However, they performed poorly on bird and mammal names, with GloVe-BAM obtaining better results than CBOW-BAM.

In order to verify our claims, we conducted one-way ANOVA tests followed by multiple comparison tests for each category. We used MAP scores as the basis of comparison as they effectively capture precision at different recall levels. Given the multiple models involved, we applied comparison methods that can provide family-wise error rates, such as Bonferroni and Dunnett’s tests [57]. For the latter test we used the highest scoring methods as the control model, i.e., CBOW and GloVe. Detailed results of our statistical significance tests are available in S1 File. Consistent conclusions were drawn based on both tests, which can be summarised as follows: (1) The performance of GloVe is not significantly better than that of CBOW, while both models significantly outperformed the remaining DSM methods on BridLife and CoL bird names (*p* < 0.01); (2) on both ITIS and CoL mammal names, GloVe was shown to perform best (*p* < 0.01); and (3) with respect to names from the PLANTS database, CBOW is superior (*p* < 0.01), while in the case of CoL plant names, there is no significant difference between the performance of CBOW and CBOW-BAM; the rest of the models, however, demonstrated significantly lower performance on both PLANTS and CoL (*p* < 0.01).

The superior performance of compositional models in the plant category can be explained by the fact that semantic variants in this category, unlike in the case of bird and mammal names, are more likely to share genus or specific epithet names with the preferred names (Table 3). This meant that the compositional model-derived vector representations of semantic variants contained common elements, thus leading to increased vector similarity. This, in turn, increased the likelihood of recognising these names as semantically related. The optimal performance obtained by compositional models was likewise observed by Thompson et al. [38] in the case of medical concepts.

The difference in performance between CBOW-BAM and CBOW on PLANTS and CoL plant names can be explained by the different proportions of names with shared components in each reference standard. Table 3 shows that about 85% of CoL plant names contain components that overlap with their semantic variants, which is higher than that for the PLANT database (about 68%). As explained above, a higher proportion of names with shared components leads to a boost in the performance of compositional models. Therefore, it is not surprising that the performance of CBOW-BAM is comparable to that of CBOW in the case of CoL plant names, but relatively worse on names from the PLANTS database.

Some observations on the highest-scoring results indicate that the applied methods generate semantically related names that are not contained in the reference standard data sets. For example, for the bird called “tree swallow”, BirdLife provides only one corresponding name, i.e., *Tachycineta bicolor*. However, the best performing method identifies the following as the five top-ranked candidates: “barn swallow”, “bank swallow”, “cliff swallow”, *Tachycineta bicolor* and *Petrochelidon pyrrhonota*. Whilst the first three names and the last one, which is the scientific name of “cliff swallow”, are not semantic variants according to BirdLife, further verification with the Cornell Lab of Ornithology’s database showed that these species are similar to “tree swallow” [58]. Another interesting example is the case of the mammal *Rhinoceros unicornis*, for which our method predicted the following five topmost candidates: “Indian rhinoceros”, *Rhinoceros indicus*, *Diceros bicornis*, *Rhinoceros sondaicus*, and “black rhinoceros”. According to ITIS, only the first two are semantic variants; the other three, of which “black rhinoceros” is a common name of *Diceros bicornis*, are not recorded as semantic variants of *Rhinoceros unicornis*. However, ARKive, a global non-profit initiative that collects information about the world’s endangered species, shows that they are closely related to *Rhinoceros unicornis* [59]. These findings demonstrate that by applying text mining techniques, we can automatically extract a richer set of related names that can potentially be used to expand lexical resources.

Currently, our methods do not handle scientific names appearing in their abbreviated forms, e.g., *A. insignis*. As discussed earlier, such abbreviations lead to ambiguity. Including them in the term inventory thus requires disambiguation, i.e., selecting the most likely correct expansion of *A. insignis* out of multiple possible ones. This task is an important step towards fully understanding textual content [60], and will be part of our future work. We finally selected all preferred species names that appear in BHL documents with a frequency of at least five, resulting in a final list of 288,562 names. The GloVe model was applied on them to construct a full inventory. For each species name in the inventory, the 20 topmost semantically related names are provided, together with their corresponding similarity scores. Furthermore, each name in the inventory has been linked to the GNI through the assignment of Global Names URIs. The term inventory is publicly available at http://metashare.metanet4u.eu/go2/bhl_inventory.

## Application to automatic query expansion

In addition to the evaluation presented above, we evaluated our term inventory from a more practical point of view. Specifically, we implemented a visual search interface incorporating our term inventory to enable automatic query expansion. One of the goals in developing this interface is to evaluate whether the term inventory can be useful in terms of increasing the number of relevant documents retrieved by a search engine. In this way, the term inventory facilitates the suggestion of terms that are semantically related to the species name supplied as a query, which are in turn appended to the query. As opposed to other automatic query expansion methods [61], the inclusion of additional terms to expand a query depends upon the user’s preferences, making our interface’s behaviour similar to that of a recommender system [62]. Another goal is to assess the usefulness of the automatically suggested semantically related terms that are uniquely available in our inventory, distinguishing it from several other taxonomies such as CoL, EoL and GBIF. With the integrated term inventory, the interface can show users not only documents matching the original query but also those that mention semantically relevant species which have, for example, shared characteristics such as habitat or taxonomic classification. For instance, as a user searches for documents pertaining to “lion”, it might be useful for him or her to find documents mentioning not only “lion” but also *Panthera leo*—the scientific name of “lion”—and “jaguar”—a big cat similarly belonging to the *Panthera* genus. In contrast, without our term inventory, the search results will include only documents that match exactly the same species, e.g., “lion” and *Panthera leo*.

### Visual search interface

**Fig 8:**
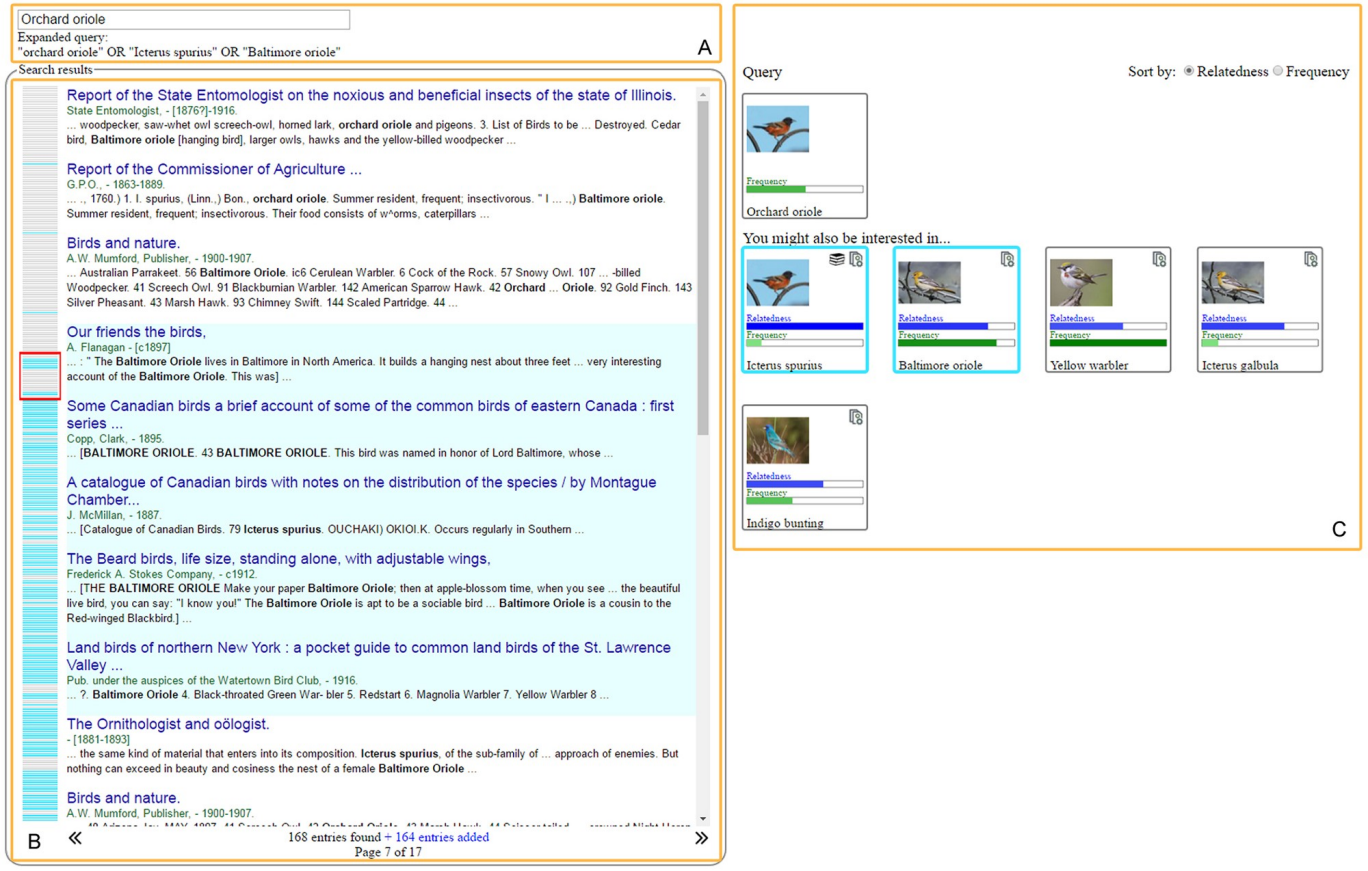
Visual search interface incorporating suggested semantically related names for query expansion. **A**- Initial and expanded query. **B**- Search result list and context viewer. The context viewer on the left-hand side shows a zoomed out view of the retrieved list. Documents retrieved according to the expanded query are shown with a light blue background. **C**- Thumbnails with suggested names for query expansion. Apart from a relevant image, each thumbnail depicts the suggested term’s frequency within BHL documents, its relatedness to the query term, and the provenance of the suggestion, i.e., our term inventory or other external resources such as CoL, EoL and GBIF.

The tool serves as a regular search interface: it retrieves documents relevant to a user-supplied query, and returns them in a list whose entries are ranked by relevance. The web-based interface is shown in Fig 8, together with a description of its main components. On the left-hand side is a context viewer (inspired by the overview+detail approach [63]) that provides an overview of the results, indicating the portion of the ranked list that the user is currently looking at. Unlike regular search interfaces, terms semantically related to the query species name are also presented on the right-hand side of the screen as thumbnails. We aimed to present the suggested terms in a simple yet informative manner. To this end, each thumbnail is displayed with two parallel indicator bars, one representing the term’s frequency in the BHL corpus and the other its relatedness to the query term. This eliminates the need to present their exact values to the user, facilitating a more intuitive visual comparison of these measures. Within the thumbnails, there are also small icons that indicate whether the name exists as a semantically related term in our term inventory, in an external resource (e.g., CoL, EoL, and GBIF) or in both. For example, in Fig 8, there are five species names returned by our interface (under the heading “You might also be interested in…”) for the query *Orchard oriole*, the first of which was suggested by both our term inventory and external resources, and the remaining four recommended by just our term inventory (as indicated by the icons at the upper right-hand corner of each thumbnail). Hovering over a thumbnail reveals detailed information about the suggested term. Images of the species retrieved from EoL (via their web services [64]) are shown for reference purposes.

Upon selection of any of the thumbnails, the terms that they correspond to become appended to the original query (with an OR operator) thus expanding it and retrieving more documents. In order to help the user assess which of the documents were retrieved as a result of expanding the query, and which ones were part of the original search result, different background colours are used both in the search result list and in the context viewer. The front-end was implemented using the JavaScript-based library D3 [65]. Meanwhile, as the interface’s underlying search engine, Apache SolR [66] was used for indexing BHL text and matching queries against them. The most current version of the interface, which is available at http://nactem.ac.uk/BHLQueryExpansion/, is built upon 2 million BHL pages indexed with the bird, mammal and plant species names used in the evaluation described in the previous section.

### User feedback

We invited 20 biologists to assess the usefulness of the interface. They were requested to explore the search system using six different scientific or vernacular species names as queries. Whilst three of the queries were pre-supplied by us, for the other three, the users were asked to enter their own preferred species names belonging to any of the following three taxonomic classes: “Aves” (birds), “Mammalia” (mammals), and “Magnoliopsida” (flowering plants). Scientific names had to be provided according to the binomial nomenclature, consisting only of the genus and specific epithet, e.g., *Lepus timidus* and *Spizella passerina*. In exploring the results of each query, we encouraged them to select a few of the terms automatically suggested by the interface, in order for them to have an appreciation of the effects of the query expansion feature.

We designed a questionnaire sheet containing eleven questions, eight of which were compulsory; the other three were optional. The eight compulsory questions consist of two subsets. The first four questions were formulated to evaluate whether the expansion of queries in a semi-automatic manner is useful or not. In particular, one of the questions aims to estimate the helpfulness of suggested species names that are not necessarily semantic variants but are semantically related, i.e., through shared habitat, geographic location or taxonomic class as in the case of “jaguar” and “lion”. The second subset of four questions sought to obtain feedback on the visual functionalities of the interface. In each question, users were asked to assess a specific functionality by rating its usefulness/helpfulness from 1 (not useful/helpful at all) to 5 (very useful/helpful). These questions are presented in detail in S2 File.

**Fig 9:**
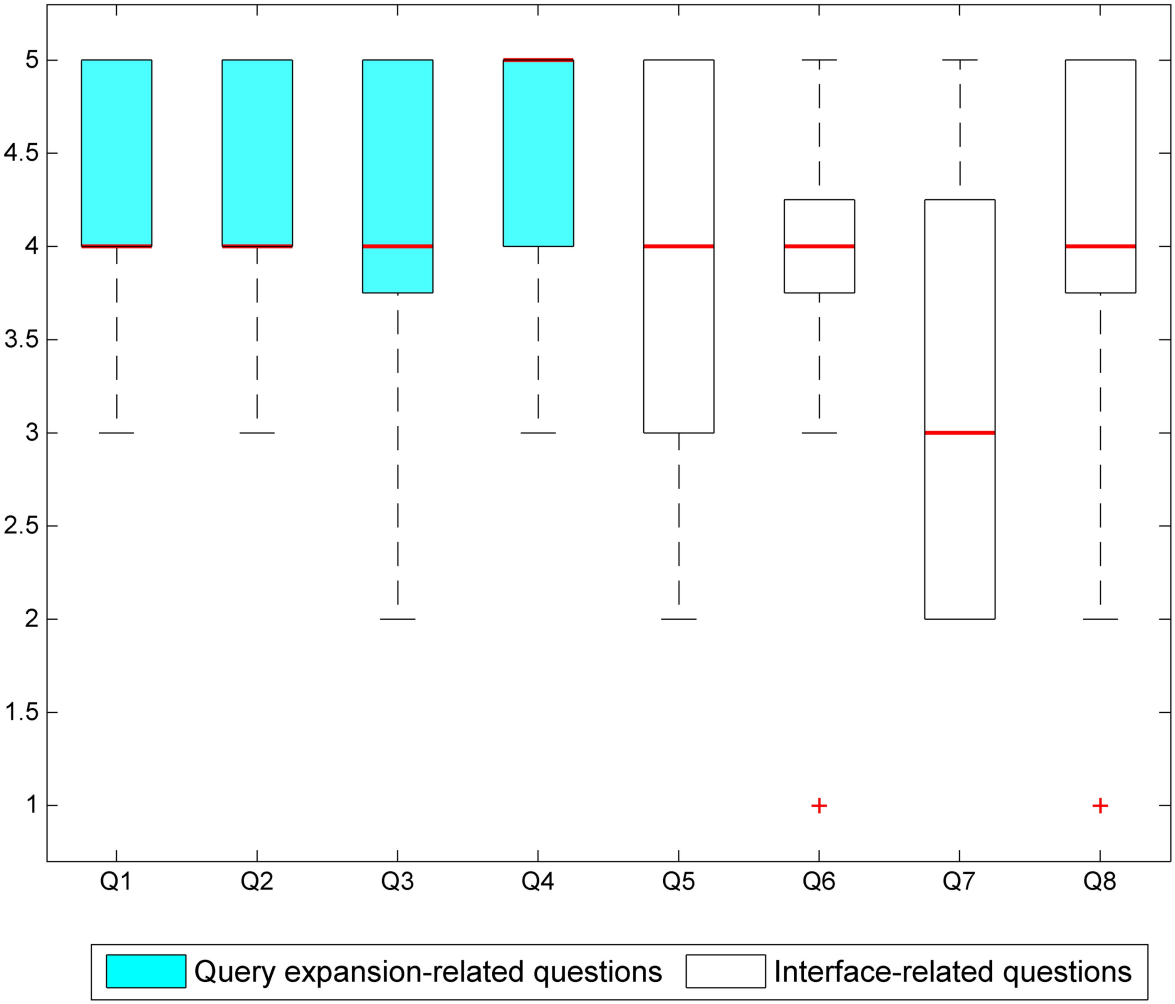
Ratings for the visual search interface given by thirteen users. The red line indicates the median rating for each question.

We collected 13 sets of responses from 20 biologists (provided for the reader’s reference as S3 File). Their ratings for the eight compulsory questions are depicted in Fig 9. We can see that the median ratings for the first four questions are consistently high, i.e., from 4 to 5, which can be generally thought of as an indication that our users find the term inventory-based query expansion functionality to be useful. Based on the responses to Question 1, most users agreed that the inventory’s suggested terms are useful. In terms of broadening the scope of search results (covered by Questions 2 and 4), responses ranged from 3 to 5, indicating that users are generally satisfied with this system feature. For Question 3, which assessed whether automatically suggested semantically related terms are useful although not all of them are exact synonyms, all of our users responded positively (with answers ranging from 3 to 5), except for one who gave a rating of 2. These responses reveal that most users share the opinion that it is helpful to be presented with suggestions of species names that are not necessarily synonyms but may be related to the original query in other ways, e.g., in terms of shared habitat, taxonomic class, or geographic location.

Meanwhile, we received diverse responses to the interface-focussed questions. Amongst our interface’s four visual features, the images (Question 5), relatedness indicator (Question 6) and different background colours (Question 8) were considered very useful, despite a few outlier ratings from some users. The users’ ratings of the frequency indicator (Question 7) show that it is deemed as relatively less useful, with a median value of 3. One reason for such lower rating could be the lack of a more detailed explanation of what the depicted frequency value stands for.

One of the optional questions in the questionnaire was aimed at gathering feedback on the features of the interface that the users liked the most. Eight of the users liked the capability of the system to automatically incorporate additional terms into the query, thus retrieving a broader set of search results. For the other users, meanwhile, what struck them the most are some of the interactive visualisations that are present in the interface, e.g., the thumbnails with images and indicator bars. We are aware, however, that the images retrieved by the EoL web service for certain species are incorrect; part of our future work will look into alternative image sources.

Based on the users’ suggestions, we enhanced the interface with two additional functionalities. Firstly, we have enabled users to perform either query expansion or query refinement, by toggling between the boolean operators OR and AND. Secondly, we addressed the issue of misspellings at the query level by incorporating string similarity methods into the interface. In this way, correctly spelt names will be suggested to users when they input misspelt ones as their initial query. Although majority of our recruited users agreed that the term inventory-driven automatic query expansion is useful, in the future we could, for instance: (1) allow the user to enter more than one species name as his/her query; (2) give the user the capability to remove any spurious results brought about by the automatically expanded query; and (3) further improve handling of misspellings, i.e., enable the capability to retrieve documents even if the names contained within their text are misspelt versions of a user’s query.

## Conclusions

We have proposed a method for automatically constructing a term inventory for biodiversity using DSMs to identify names which are semantically related to any given species name. Firstly, models were trained on the English subset of BHL. Evaluation of their performance according to four different metrics reveals that prediction-based DSMs performed significantly better than count-based DSMs, with the GloVe model obtaining the best performance in most cases. We also observed that compositional DSMs perform better than non-compositional ones in the case of multi-word terms sharing component words. The resulting term inventory contains more than 288,000 scientific and vernacular names of species. For each species name, its 20 topmost semantically related terms are provided, together with their respective similarity scores. Upon manual inspection of some sample terms, we observed that our term inventory contains semantically related names that are not yet recorded in other published dictionaries and taxonomic resources.

We demonstrated one application of the term inventory, i.e., semi-automatic search query expansion. To evaluate the effect of query expansion based on automatically suggested species names, we developed a system that incorporated our term inventory into a visual search interface. Upon user query input, the interface suggests a list of semantically related terms. The selection of any of these suggested terms expands the original query, thus broadening the scope of the search results. Thirteen experts evaluated the usefulness of the search interface’s functionalities, most of whom agreed that the semantically related terms suggested by our term inventory are useful.

Our work has demonstrated that the text mining-based construction of a terminological inventory is feasible, especially for domains in which large amounts of textual data are available. We showed how the resulting term inventory could potentially enhance search systems, by facilitating the automatic expansion of search queries.

Our methods were developed with the aim of capturing semantically related names, and are most useful in the context of search and query expansion. Adapting our methods to capture only strict-sense synonyms for the purpose of contributing to resources that contain only such type of synonyms (as in the case of the Global Names Index) could form part of our future work. We envision that this type of curation can be carried out in a semi-automatic manner, in that a taxonomist validates the recommendations supplied by the algorithm, alongside any textual evidence from the literature that was used to infer the association. Although manual effort will still be required, we could expect significant benefits in terms of (1) reduced curation time, as only a small sample of semantic variants will need to be validated per species name; and (2) the high coverage that our methods obtain by mining information from large amounts of biodiversity textual content.

## Supporting information

S1 Text Reference standard data sets used in the evaluation of distributional semantic models. (TXT)

S1 File Results of the statistical tests. (XLSX)

S2 File Questionnaire for gathering user feedback on our visual search interface. (DOCX)

S3 File Responses to the questionnaire on our visual search interface. (XLSX)
