# Supplementary material for: Constructing a biodiversity terminological inventory
Source: PLoS One. 2017 Apr 17;12(4):e0175277. doi: 10.1371/journal.pone.0175277 (PMC5393592; doi:10.1371/journal.pone.0175277)
Supplement: S2 File — (DOCX) [file pone.0175277.s003.docx]

**Questionnaire Sheet**

According to your experience with the query expansion interface, please rate your answer in a range from very useful/helpful to not useful/helpful at all for the following questions.

1. In general, did you find the suggested terms useful for this type of search?

| ○ | ○ | ○ | ○ | ○ |
| --- | --- | --- | --- | --- |
| 1 (Not useful at all) | 2 | 3 | 4 | 5 (Very useful) |

1. Do you think that the suggested additional species names helped you retrieve documents that you may not have found otherwise?

| ○ | ○ | ○ | ○ | ○ |
| --- | --- | --- | --- | --- |
| 1 (Not helpful at all) | 2 | 3 | 4 | 5 (Very helpful) |

1. Was it helpful to be presented with suggestions of species names that are not necessarily synonyms but may be related in terms of shared habitat, taxon or geographic location? For example, suggesting ‘jaguar’ when searching for ‘lion’.

| ○ | ○ | ○ | ○ | ○ |
| --- | --- | --- | --- | --- |
| 1 (Not helpful at all) | 2 | 3 | 4 | 5 (Very helpful) |

1. Expansion of the original query with additional terms broadens the scope of the search results. Did you find this useful?

| ○ | ○ | ○ | ○ | ○ |
| --- | --- | --- | --- | --- |
| 1 (Not useful at all) | 2 | 3 | 4 | 5 (Very useful) |
|  |  |  |  |  |

1. Were the illustrated images helpful in the selection of suggested terms?

| ○ | ○ | ○ | ○ | ○ |
| --- | --- | --- | --- | --- |
| 1 (Not helpful at all) | 2 | 3 | 4 | 5 (Very helpful) |

1. Was the relatedness indicator for suggested terms helpful?

| ○ | ○ | ○ | ○ | ○ |
| --- | --- | --- | --- | --- |
| 1 (Not helpful at all) | 2 | 3 | 4 | 5 (Very helpful) |

1. Was the frequency indicator for suggested terms helpful?

| ○ | ○ | ○ | ○ | ○ |
| --- | --- | --- | --- | --- |
| 1 (Not helpful at all) | 2 | 3 | 4 | 5 (Very helpful) |

1. Were the different background colours (white and light blue for initial and augmented search results, respectively) useful in identifying the additionally retrieved documents?

| ○ | ○ | ○ | ○ | ○ |
| --- | --- | --- | --- | --- |
| 1 (Not useful at all) | 2 | 3 | 4 | 5 (Very useful) |

**Optional**

We would appreciate if you can give us your comments on the interface.

1. What would you suggest in order to improve the interface?

1. What did you like the most from the interface?

1. Other comments (if any)
